# Job satisfaction and associated factors among health care providers at public health institutions in Harari region, eastern Ethiopia: a cross-sectional study

**DOI:** 10.1186/s13104-015-1368-5

**Published:** 2015-09-01

**Authors:** Ayele Geleto, Negga Baraki, Gudina Egata Atomsa, Yadeta Dessie

**Affiliations:** Department of Public Health, College of Health and Medical Sciences, Haramaya University, Haramaya, Harar, Ethiopia; Department of Environmental Health Science, College of Health and Medical Sciences, Haramaya University, Haramaya, Harar, Ethiopia

**Keywords:** Job satisfaction, Motivation, Performance, Health care worker

## Abstract

**Background:**

Human factor is the primary resource of health care system. For optimal performance of health care system, the workforce needs to be satisfied with the job he/she is doing. This research was aimed to assess the level of job satisfaction and associated factors among health care providers at public health institutions in Harari region, Eastern Ethiopia.

**Method:**

Health facility based cross-sectional study was conducted among 405 randomly selected health care providers in Harari regional state, Eastern Ethiopia. Data were collected by self-administered structured questionnaires. Epidata Version 3.1 was used for data entry and analysis was made with SPSS version 17. Level of job satisfaction was measured with a multi item scales derived from Wellness Council of America and Best Companies Group. The average/mean value was used as the cutoff point to determine whether the respondents were satisfied with their job or not. Multivariable logistic regression was used to analyze data and odds ratio with 95 % CI at P ≤ 0.05 was used to identify associated factors with level of job satisfaction.

**Results:**

Less than half 179 (44.2 %) of the respondents were satisfied with their job. Being midwifery in profession [AOR = 1.20; 95 % CI (1.11–2.23)], age less than 35 years [AOR = 2.0; 95 % CI (1.67–2.88)], having good attitude to stay in the same ward for longer period [AOR = 3.21; 95 % CI (1.33, 5.41)], and safe working environment [AOR = 4.61; 95 % CI (3.33, 6.92)] were found were found to be associated with job satisfaction.

**Conclusions:**

Less than half (44.2 %) of the respondents were satisfied with their current job. Organizational management system, salary and payment and working environment were among factors that affects level of job satisfaction. Thus, regional health bureau and health facility administrators need to pay special attention to improve management system through the application of a health sector reform strategy.

## Background

Human factor is the most critical resource for any organizations. It organizes and utilizes other resources for the production of the intended outputs. For the optimum performance the workforce needs to be regularly motivated through either financial or non financial incentives to get satisfied to their work [1]. Job satisfaction can be defined as a pleasurable or positive emotional state, resulting from the appraisal of one’s job or job experiences [2]. In health sector, job satisfaction is highly associated with quality of services and patient satisfaction [3].

Job satisfaction is believed to be a key factor that it influences performance of individuals and organizations. Dissatisfied work force has a negative impact on performance of the facilities. Moreover, it is a pushing factor for migration of health workers, both from rural areas to the cities and out of the country [4]. It is therefore an important effort of human resources management in the health sector to strengthen the motivation of health workers [5]. Researchers have observed that dissatisfied employees, if remained in the organization, may involve in counter-productive activities such as theft, poor service, destructive rumors and sabotage of equipment. Employees’ job dissatisfaction gives rise to high level of turnover intention which ultimately leads to actual workers turnover [6].

There are a number of interrelated factors that determine level of job satisfaction of health work force. These include unsatisfactory working conditions characterized by heavy workloads, lack of professional autonomy, poor supervision, long working hours, unsafe workplaces, inadequate career structures, poor remuneration/unfair pay, poor access to needed supplies and limited or no access to professional development opportunities and internal and international migration of workers determine the motivation of health work force [7, 8].

Despite the fact that, human power is the back bone for the provision of quality health care and professional job satisfaction earns high dividends [9], limited studies, that address job satisfaction of health workers, have been undertaken in Ethiopia. Job satisfaction of health workers in developing countries in general and in Ethiopia in particular is very low. A study conducted in Jimma University specialized hospital, southwest Ethiopia, in 2009 among 127 health workers indicated that only 41.4 % were satisfied with their job [11]. So far, no study has been undertaken in Harari Region on job satisfaction of health workers even though there is high turnover rate of health professional in the region.

The main aim of this study is to assess level of job satisfaction and its associated factors among health care providers working at public health institutions in Harari Region, Eastern Ethiopia.

## Methods

### Study design and setting

Health facility based quantitative cross-sectional study was conducted in Harari region. Harari Region is one of the nine regional states of Ethiopia. The region is found 510 km far from Addis Ababa, the capital city of Ethiopia, to the east. The region is divided into nine districts with three of them are rural and six are urban. The urban districts are sub divided into nineteen kebeles (the lowest administrative division in the country), and the rural districts are sub divided into seventeen peasant associations (which is equivalent to kebele in urban case). The region has a total population of 205,000 of which urban population comprises of 111,052 (54.8 %). Health service coverage of the region is 100 %. There are two public hospitals and eight public health centers, with 562 health care providers, in the region [10]. This study was conducted from February to March 2013 among health care providers working in all available public health institutions.

### Participants

Randomly selected health care providers, who were willing to complete the survey questionnaire, were included into the study with the exclusion of newly recruited health care providers (those working for less than 6 months at data collection period). Those who are not working in curative service (clinical setup) were also excluded from the study.

### Sample size and sampling procedures

To estimate the sample size a single population proportion formula, [n = (Z α/2)^2^ p (1 − p)/d2], was used. 41.4 % job satisfaction among health care providers, the result of a study conducted in Jimma University Specialized hospital in 2009 [11], was used. 95 % confidence level with margin of error 5 % (d = 0.05) and non-response of 10 % were considered. Therefore the final sample size calculated was 420. The sample size was proportionally allocated to each health institution based on the number of health care providers working in the respective health facilities. Then health care providers were again classified into different professional categories. The respective samples for specific profession were drawn from those specific professions by simple random sampling method and the sample size that was drawn from a specific profession depends on the number of individuals in a specific profession. A registered list of health professionals found at each health institution was used as sampling frame. Regarding health facilities, all available public hospitals and public health centers were included into the study.

### Measurements

The outcome variable of this study is level of job satisfaction of health care providers. Assessment of jab satisfaction was performed by using multi-dimensional job satisfaction scales. The scale was taken from Wellness Council of America (WELCOA) and Best Company Group (BCG) [12]. The job satisfaction questionnaire contained 36 items divided into eight main domains which include: leadership and planning, institutional culture and communication, employee’s role, pay and benefit, work environment, training and development, relationship with supervisor and co-worker relationship. Each domain has satisfaction subsidiary scales which were measured by Likert scale from highly disagree (1) to highly agree (5). The overall job satisfaction score was estimated by taking the average score of all the subscales.

To measure level of job satisfaction of each individual, the mean (average) value of all domains was calculated. Mean value of domains was taken as a cut point value to determine whether a health worker was satisfied to his/her job or not. As a result, health workers for whom score was below mean were considered as dissatisfied and those with mean and above were regarded as satisfied [13].

The independent variables of the study include age, marital status, religion, profession, working unit (ward), effect of staying in the same ward for longer time and motivation status of health workers.

### Data collection and analysis

Data were collected using pre tested self-administered questionnaires. The questionnaires were adapted from standardized questionnaire by Wellness Council of America (WELCOA) and Best Companies Group (BCG) [12] and modified to the context of the study area. The questionnaires were then translated to the local language, “*Amharic”,* for data collection. Then it was pretested on 20 health care providers with different profession. All tools were revised and finalized after pretesting. The questionnaires were enclosed in postal envelope and were distributed to the selected respondents to be filled overnight when they were not busy. Completed questionnaire was collected by data collectors on the very immediate morning and submitted to investigators. Data collectors were health professionals from Haramaya University teaching staff. They were selected depending on their experience in the field of data collection. They were given training before data collection was started. Completed questionnaires were checked every day by principal investigator and supervisors.

Data were entered to EpiData Version 3.1 and exported to SPSS version 17 statistical software for analysis. Descriptive statistics was used to summarize the data and the results were presented using frequency tables, mean, standard deviation and percentages. Multivariate logistic regression analysis was employed to control confounders between variables. Crude Odds ratio with 95 % CI was used to determine presence of association between explanatory variables and job satisfaction of respondents. The degree of association between dependent and independent variables was measured using adjusted odds ratio with 95 % confidence interval at a significance level of ≤ 0.05.

### Ethical considerations

The study secured ethical clearance from the Institutional Research Ethics Review Committee (IRERC) of College of Health and Medical Science, Haramaya University. A letter explaining about the purpose, method and anticipated benefit and risk of the study was attached to each questionnaire. Furthermore, the data collector informed the respondents on the benefit and risk of the study. All the study participants were explained about their full right to refuse or withdraw from part or all of the study. Written consent was obtained from each participant and confidentiality of the study subjects was maintained.

## Results

### Socio-demographic characteristics of the respondents

A total of 405 health care providers with various professions completed the survey questionnaires from the proposed 420, providing a response rate of 96.43 %. Among the respondents 225 (55.6 %) were females. Majority of the respondents 154 (38 %) were Amhara by ethnicity, and 174 (43 %) were Muslim by religion. The mean (±SD) age of the respondents was 30.04 (±7.502) year. About half of the study participants 207 (51.1 %) were married, and 218 (53.8 %) of them were earning a salary of less than 2000 ETB per month (which corresponds to ~104$) 1 USD is equivalent 19.2 ETB during the study period. Majority 182 (44.9 %) of the respondents were nurses while specialists accounted only for 18(4.4 %), (Table 1).

**Table 1:** Socio-demographic characteristics of participants (n = 405), HNRS, 2013

Variables	Frequency (Percent)
Sex	
Male	180 (44.4)
Female	225 (55.6)
Age	
<35	303 (74.8)
35 and above	102 (25.2)
Ethnic group	
Oromo	136 (33.6)
Amhara	154 (38.0)
Tigray	24 (5.9)
Harari	68 (16.8)
Somali	3 (0.7)
Others	20 (4.9)
Religion	
Muslim	174 (43.0)
Orthodox	153 (37.8)
Protestant	67 (16.5)
Others	11 (2.7)
Marital status	
Single	166 (41.0)
Married	207 (51.1)
Divorced	11 (2.7)
Widowed	18 (4.4)
Separated	3 (0.7)
Profession	
Specialists	18 (4.4)
General practitioner	38 (9.5)
Health officers	33 (8.3)
Nurses	182 (44.9)
Midwifes	28 (6.9)
Pharmacy professionals	51 (12.4)
Laboratory professionals	37 (9.2)
Others*	18 (4.4)
Salary	
<2000 birr	218 (53.8)
2001–3000 birr	118 (29.2)
3001–4000 birr	62 (15.3)
>4001 birr	7 (1.7)

* Others health care providers include X-ray technicians, physiotherapists and psychiatrists

*HNRS* Harari National Regional State

### Job satisfaction of health professionals

Overall, less than half 179 (44.8 %) of the respondents were satisfied with their job. With regard to each domains, nearly half 211 (52.1 %) of the participants reported that they were satisfied with their role at their institution. Two hundred nine (51.6 %) were satisfied to their relationship with their co-workers. However, only less than half of the study participants, 148 (36.5 %) were satisfied with pay and benefit package of their respective institutions. Level of satisfaction to relationship with supervisor was 163 (40.2 %). Training and development 165 (41.4 %), work environment 178 (43.9 %) and leadership and planning of the organization 175 (43.2 %) were among factors for which less than half of participants were satisfied (Table 2).

**Table 2:** Satisfaction level among participants by job satisfaction domains (n = 405), HNRS, 2013

Job satisfaction domains	Level of satisfaction	
	Satisfied	Dissatisfied
	N (%)	N (%)
Leadership and planning	175 (43.2)	230 (56.8)
Culture and communications	180 (44.6)	225 (55.4)
Employee’s role	211 (52.1)	194 (47.9)
Work environment	178 (43.9)	227 (56.1)
Relationship with supervisor	163 (40.2)	242 (59.8)
Training and development	165 (41.4)	237 (58.6)
Pay and benefits	148 (36.5)	257 (63.5)
Coworker relation ship	209 (51.6)	196 (48.4)
Overall satisfaction	179 (44.2)	226 (55.8)

*HNRS* Harari National Regional State

Satisfaction according to professional background of the respondents showed that more than half of laboratory workers (54.1 %) and midwifes (53.6 %) were satisfied to their job. However, the highest dissatisfaction lies among nursing staffs (72.1 %) followed by health officers (60.6 %). 52.6 % of general practitioners were also dissatisfied to their job while half of the specialists were satisfied (Table 3).

**Table 3:** Distribution of job satisfaction by profession of participants, HNRS, 2013

Profession	Level of satisfaction	
	Satisfied	Dissatisfied
	N (%)	N (%)
Specialists (n = 18)	9 (50)	9 (50)
General practitioner (n = 38)	18 (47.4)	20 (52.6)
Health officer (n = 33)	13 (39.4)	20 (60.6)
Nurse (n = 182)	69 (37.9)	113 (62.1)
Midwife (n = 28)	15 (53.6)	13 (46.4)
Pharmacy (n = 51)	22 (43.2)	29 (56.8)
Lab (n = 37)	20 (54.1)	17 (45.9)
Others (n = 18)	13 (72.2)	5 (27.8)
Total	179 (44.2)	226 (55.8)

### Factors associated with job satisfaction

Multivariate logistic regression analysis showed that age of respondents, profession, level of satisfaction with staying in the same working unit for longer period, comfortable working area that is free of hazard, future intention, satisfaction to motivation system and current position were significantly associated with job satisfaction. Health care workers in the age group of less than 35 years were 2 time more likely satisfied with their job compared to those 35 years and more [Adjusted OR = 2.0; 95 % CI (1.67, 2.88)]. Midwifes [Adjusted OR = 1.2; 95 % CI (1.11, 2.23)] were more likely satisfied with their job than other health workers. The odd of satisfaction was 3.21 fold higher among health worker who were satisfied to their stay in the same ward for longer period than those who dissatisfied to it [Adjusted OR = 3.21; 95 % CI (1.33, 5.41)] (Table 4).

**Table 4:** Multivariable logistic regression analysis of factors associated with job satisfaction of participants, HNRS, 2013

Variables	Level of satisfaction		COR (95 % CI)	AOR (95 % CI)
	Satisfied	Dissatisfied		
Age				
<35	172 (42.5)	129 (31.8)	1.89 (1.22, 2.11)	2.0 (1.67, 2.88)
35 and above	43 (10.6)	61 (15.1)	1.00	1.00
Profession				
Specialist	9 (50)	9(50)	1.00	1.00
General practitioner	18 (47.4)	20 (52.6)		
Health officer (HO)	13 (39.4	20 (60.6)		
Nurse	69 (27.9 %)	113 (72.1)		
Midwife	15 (53.6)	13 (46.4)	1.15 (1.01, 1.78)	1.20 (1.11, 2.23)
Pharmacy	22 (43.2)	29 (56.8)		
Laboratory	20 (54.1)	17 (45.9)	1.17 (1.01, 1.81)	
Others**	13 (72.3)	5 (27.7)		
Salary				
<2000 birr	67 (16.5)	151 (37.3)	0.34 (0.15, 0.54)	
2000 birr and above	105(25.9)	82 (20.3)	1.00	
Health institution				
Health center	59 (14.6)	47 (11.6)	1.87 (1.19, 2.93)*	
Hospital	120 (29.6)	179 (44.2)	1.00	
Satisfaction with stay				
Yes	136 (33.6)	105 (25.9)	3.64 (1.17, 4.76)*	3.21 (1.33, 5.41)
No	43 (10.6)	121 (29.9)	1.00	1.00
Safe environment				
Yes	116 (28.7)	80 (19.8)	3.36 (2.12, 4.68)*	
No	63 (15.5)	146 (36.0)	1.00	
Free of hazard				
Yes	113(27.9)	64 (15.8)	4.33 (3.46, 5.86)*	5.10 (3.82, 7.99)*
No	66 (16.3)	162 (40)	1.00	1.00
Comfortable				
Yes	101 (24.9)	50 (12.3)	4.55 (3.25, 6.69)*	4.61 (3.33, 6.92)*
No	78 (19.3)	176 (43.5)	1.00	1.00
Med. equipment				
Adequate	91 (22.5)	73 (18.1)	2.16 (1.23, 3.89)*	
Inadequate	88 (21.7)	153 (37.7)	1.00	
Essential drug				
Adequate	84 (20.7)	50 (12.3)	3.11 (1.86, 5.10)*	
Inadequate	95 (23.5)	176 (43.5)	1.00	
Future intention				
To stay	110 (27.2)	92 (22.7)	2.32 (1.55, 3.46)*	3.01 (1.33, 6.79)
To leave	69 (17.0)	134 (33.1)	1.00	1.00
Essential drug				
Adequate	84 (20.7)	50 (12.3)	3.11 (1.86, 5.10)*	
Inadequate	95 (23.5)	176 (43.5)	1.00	

*HNRS* Harari National Regional State

* p value less than 0.001

** Others include X-ray technicians, physiotherapists and psychiatrists

## Discussion

Job satisfaction of health care providers plays a great role in providing a quality healthcare. It helps to achieve MDGs, especially in third world country including Ethiopian, where most of the indicators are lagging behind [11]. The findings of our study indicated that less than half (44.2 %) of the study participants were satisfied with their job. It is inconsistent with the finding of a study conducted in Mainland China among professional nurses where more than half (53.7 %; n = 275) were satisfied with their job [14]. The result of this study is also inconsistent with the findings of the study conducted in Ireland in 2009 among 766 community pharmacists which indicated, 57 % of respondents, were satisfied with their job [15]. Similar, but a little higher, finding was reported in Ethiopia, where only 41.4 % of health care providers working at Jimma specialized University hospital, were satisfied with their job [11].

In our study, only 36.5 % of participants were satisfied to payment and benefit system of their respective organization. This finding is similar to the findings of Pascal study where pay and benefit accounted for the lowest among other satisfying factors [4, 16]. Naturally, personnel are sensitive to salary issues because of their impact on living standards. Thus, a low level of satisfaction payment is a common problem among all studies of employees’ satisfaction. This might be due to high salary expectation of employees and comparing government payment with private and NGO payment which is higher.

The result of our study showed that 56.1 % of the respondents were dissatisfied with working environment. It is similar to the findings of a study conducted in Iran. Working conditions must be suitable for personnel needs and their expectations. This includes factors such as lighting, heating, air circulation and noise [17].

Satisfaction with leadership style and communication modality was also low (44.3 %), which is similar to Hong Lu’s study [14]. Management types and communication possibilities with superiors are important to job satisfaction. Consulting with health care providers and giving importance to their decisions and feelings will provide greater satisfaction. Democratic leadership and multidirectional communication can create good attitude toward achieving common organizational goal. It is far less expensive and more effective to give recognition to an employee for a well done job to make them satisfied with their job [14].

In our study satisfaction to training and development opportunity is low. Only 41.4 % of the respondents were satisfied with the opportunity to training and development. Those who had attended some form of training in the preceding 12 months were more likely to have higher satisfaction scores when compared to those who had never attended any type of training. The finding is similar with the finding of a study conducted in Iran, which showed that in-service training could be a motivating factor for health workers rather than just a focus on higher wages [18]. Refreshment of skill may build up the confidence of health care providers and increases their creativity. It also motivates individuals as it is highly related with benefit and reliving workers from work tension. The possible explanation given why health workers dissatisfied with this aspect may be unfair screening of individuals for training, repeated selection of an individual for training.

Satisfaction with coworker relationship in this study was relatively high (51.5 %) which is similar to findings from Pascal study [4]. Good relations among colleagues and having the support of superiors and subordinates generally create a feeling of satisfaction. Research on social networks has also shown that social support from coworker networks serves as a resource that affects job satisfaction [18]. Slightly more than half of the respondents (52.1 %) were also satisfied with their role in their respective institution. The finding is in line with a study result of Hong Lu. Understanding of one’s own role in an organization was enjoyable; provide sense of responsibility and ownership which could significantly increase job satisfaction. This may reflect compatible demands from health care providers and health care managers resulting in clear and sufficient information about working responsibilities. Health care providers and managers may have similar values and principles, thus reducing the potential for role conflict [14].

The overall satisfaction patterns showed interesting variations that the mean satisfaction scores varied by type of profession. Among the health workers, laboratory professionals were highly satisfied when compared to other health workers. This could be attributed to relatively good working environment in relation to availability of medical equipment and working space where basic laboratory instruments were fulfilled. Interestingly midwifes were appeared to be more satisfied when compared to other health workers. This could be attributed to the fact that there were frequent training opportunity and professional allowance for midwife professionals. This finding is consistence with Wilbroad study in Zambia [19].

### Limitation of the study

The study was suffered from the usual limitation of a cross sectional study. The sample size was relatively small and restricted to health care providers in Harari Region. Consequently the findings may not be generalized to health workers in other districts or other cultural contexts. The tool used in this study was adapted from standardized tool appropriate in the context of developed country which may not suitable of the case of developing country.

## Conclusion

More than half of health professionals working in public health institutions in Harari region were dissatisfied with their job. Payment and benefit, lack of training and development, relationship with organizational leaders, poorly designed working environment and organizational culture and communication were the major factors that decrease the satisfaction level of the health workers n. Thus, policy makers and health service managers of the region, if they really need to deliver quality health care, need to pay special attention to improve management system through the application of a health sector reform strategy.

## Abbreviations

AIDS: acquired immune deficiency syndrome
BCG: Best Companies Group
ETB: Ethiopian birr
HIV: human immunodeficiency virus
HRHB: Harari Regional Health Bureau
HNRS: Harari National Regional State
HU: Haramaya University
IRERC: Institutional Research and Ethical Review Committee
MD: medical doctor
MDG: millennium development goal
NGO: non governmental organization
OPD: out patient department
WELCOA: Wellness Council of America
WHO: World Health Organization
